# Blockade of MCP-1/CCR4 signaling-induced recruitment of activated regulatory cells evokes an antitumor immune response in head and neck squamous cell carcinoma

**DOI:** 10.18632/oncotarget.9265

**Published:** 2016-05-10

**Authors:** Wei Sun, Wei-Jin Li, Fan-Qin Wei, Thian-Sze Wong, Wen-Bin Lei, Xiao-Lin Zhu, Jian Li, Wei-Ping Wen

**Affiliations:** ^1^ Department of Otorhinolaryngology-Head and Neck Surgery, The First Affiliated Hospital, Sun Yat-Sen University, Guangzhou, China; ^2^ Institute of Otorhinolaryngology-Head and Neck Surgery, Sun Yat-Sen University, Guangzhou, China; ^3^ Department of Otorhinolaryngology-Head and Neck Surgery, The Sixth Affiliated Hospital, Sun Yat-Sen University, Guangzhou, China; ^4^ Department of Surgery, Queen Mary Hospital, The University of Hong Kong, Hong Kong, China

**Keywords:** CCR4, Treg, antitumor immunity, prognosis, head and neck squamous cell carcinoma

## Abstract

FoxP3^+^ regulatory T (Treg) cells have diverse functions in the suppression of antitumor immunity. We show that FoxP3^hi^CD45RA^−^CD4^+^ Treg cells [activated Treg (aTreg) cells] are the predominant cell population among tumor-infiltrating FoxP3^+^ T cells, and that high aTreg cell-infiltrating content is associated with reduced survival in patients with head and neck squamous cell carcinoma (HNSCC). *In vitro* studies have demonstrated that aTreg cells can suppress tumor-associated antigen (TAA) effector T cell immune responses in HNSCC. Moreover, C-C chemokine receptor 4 (CCR4) was specifically expressed by aTreg cells in the peripheral blood of HNSCC patients. Using a RayBiotech human chemokine antibody array, we showed that monocyte chemoattractant protein-1 (MCP-1), an endogenous CCR4-binding ligand, was specifically upregulated in the HNSCC microenvironment compared to the other four CCR4-binding ligands. Blocking MCP-1/CCR4 signaling-induced aTreg cell recruitment using a CCR4 antagonist evoked antitumor immunity in mice, and lead to inhibition of tumor growth and prolonged survival. Therefore, blocking aTreg cell trafficking in tumors using CCR4-binding agents may be an effective immunotherapy for HNSCC.

## INTRODUCTION

Head and neck squamous cell cancer (HNSCC) is the sixth most common type of cancer [1]. Despite new treatment modalities that can improve overall quality of life, survival rates have not improved in the past 30 years [2]. Progression and recurrence of HNSCC and other malignancies are associated with severe immune dysfunction [3, 4]. Tumor-infiltrating FoxP3^+^CD25^+^CD4^+^ regulatory T (Treg) cells can promote local tumor growth through exerting immunosuppressive activities against tumor-associated antigen (TAA) T cell responses [3, 5–11]. Thus, tumor-infiltrating Treg cells are a major obstacle in cancer immunotherapy [9, 10, 12–14].

Studies involving depletion of Tregs have shown that depletion of CD25^+^ T cells enhances antitumor immune responses [15, 16]. However, similar studies have failed to demonstrate these effects [17–19]. The apparent confusion regarding the role of Treg cells in suppressing antitumor immune responses might be explained as follows. Activated, nonsuppressive CD4^+^ T cells also express CD25, and interleukin (IL)-2 produced by these cells is required for CD8^+^ cytotoxic T cell expansion. Depletion of CD25^+^ T cells may also reduce the number of activated CD4^+^ T cells [17]. Additionally, in some animal studies, total depletion of Treg cells has triggered autoimmunity [20–22]. Thus, we aimed to block Treg cell trafficking and accumulation in the tumor microenvironment, which could provide an attractive therapeutic strategy against immune suppression.

The Sakaguchi group demonstrated that human FoxP3^+^CD25^+^CD4^+^ T cells can be divided into the following three functionally distinct subsets on the basis of CD45RA, Foxp3, and CD25 expression: (*i*) FoxP3^lo^CD45RA^+^CD4^+^ resting Treg (rTreg) cells, which are CD25^++^, (*ii*) FoxP3^hi^CD45RA^−^CD4^+^ activated Treg (aTreg) cells, which are CD25^+++^, and (*iii*) cytokine-secreting FoxP3^lo^CD45RA^−^CD4^+^ non-Treg (nTreg) cells, which are CD25^++^ [23]. We recently reported an increase in aTreg cells, which are highly suppressive, and a decrease in rTreg cells in HNSCC patients. Although there was also an increase in nTreg cells, these cells did not have immune-suppressive activities [24, 25]. Thus, we postulated that preventing the accumulation of aTreg cells within a tumor could evoke and enhance antitumor immune responses.

Preventing the accumulation of circulating aTreg cells in a tumor site requires an understanding of the mechanisms that mediate the recruitment of these cells within HNSCC tissues. Chemokine receptors are one class of proteins that could direct Treg cell migration to tumor sites by sensing a specific chemokine milieu [26].

In this study, we evaluated the clinical importance of tumor-infiltrating aTreg cells in HNSCC patients. We found that the C-C chemokine receptor (CCR) 4 was predominantly expressed on aTreg cells compared to rTreg and nTreg cells, FoxP3^−^CD4^+^ T cell subsets, and other types of immune cells, indicating that CCR4-binding agents could be used to selectively block aTreg cell trafficking. Additionally, we showed that MCP-1/CCR4 signaling induced aTreg cell trafficking in tumors. Blockade of aTreg cell trafficking augmented the antitumor immune response thereby preventing xenograft tumor growth and prolonging mouse survival.

## RESULTS

### Accumulation of aTreg cells in HNSCC tissue accelerates disease progression and predicts poor survival

**Figure 1:**
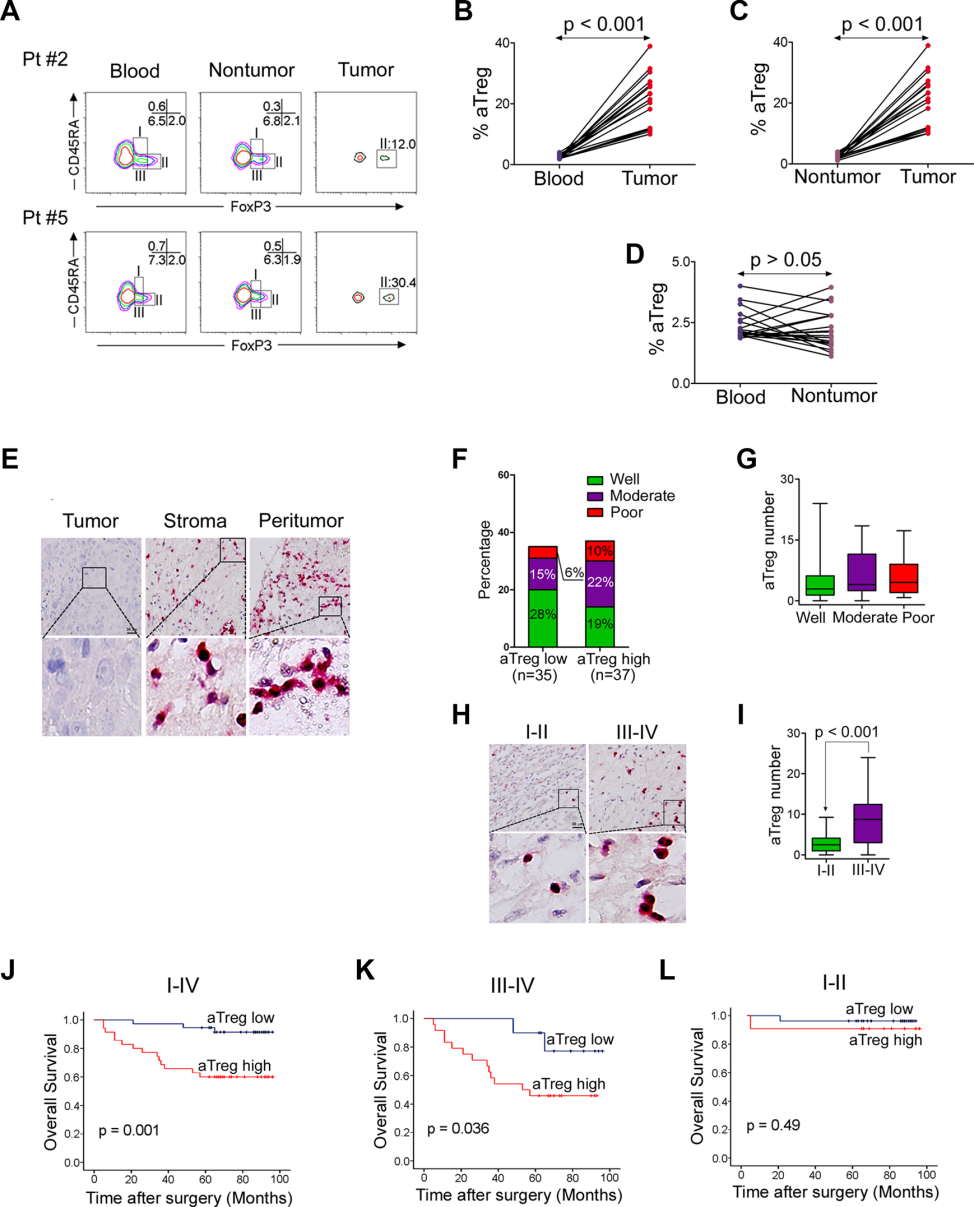
Phenotype and clinical implications of tumor-infiltrating Treg cells (**A–D**) Predominant infiltration of aTreg cells into HNSCC tissues. (A) CD4^+^ T cells from peripheral blood, adjacent nontumor sites, and tumor sites were fractionated into subpopulations based on the expression of CD45RA and FoxP3. The frequency of CD4^+^ T cells in each fraction was analyzed. Data from two representative patients are shown. (B–D) Comparison of the frequency of aTreg cells within peripheral blood, adjacent nontumor sites, and tumor sites (*n* = 19). (**E**) Immunohistochemistry of aTreg cells in LSCC samples (*n* = 72). Representative images showing staining of aTreg cells within the tumor, stroma, and peritumor sites are shown. Brown: FoxP3; Red: CD25. Scale bars: 30 μm. The lower panels are magnified images of the boxed area in the corresponding upper panel. (**F**) Comparison of the percentage of differentiation of tumors with low levels of aTreg cell infiltration compared to those with high levels. (**G**) Box plot showing quantitative evaluation of aTreg cell infiltration. Statistical differences between the three groups were analyzed using Kruskal-Wallis tests. (**H–I**) The level of aTreg cell infiltration in patients with early-stage tumors was lower than the level in patients with late-stage tumors. (H) Representative images showing staining of aTreg cells within tumor tissues from patients with early-stage (I and II) and late-stage (III and IV) tumors. (I) Statistical differences between the two groups were analyzed using Mann-Whitney *U*-tests. (**J–L**) Accumulation of tumor-infiltrating aTreg cells predicts poor survival in patients with LSCC. Kaplan-Meier curve for overall survival according to the number of tumor-infiltrating aTreg cells in 72 LSCC patients at (J) stages I–IV, (K) stages III and IV only, or (L) stages I and II only. Samples were divided into two groups based on the level of aTreg cell infiltration.

We first analyzed the prevalence of tumor-infiltrating FoxP3^hi^CD45RA^−^CD4^+^ aTreg cells in 19 clinical HNSCC specimens. The majority of tumor-infiltrating FoxP3^+^ T cells were FoxP3^hi^CD45RA^−^CD4^+^ aTreg cells (Fr. II) (Figure 1A). Moreover, the frequency of these aTreg cells in tumors was significantly higher than in PBMCs and adjacent nontumor tissues (*P* < 0.001) (Figure 1B–1D).

Because the various subtypes of HNSCC have different etiologies and survival rates, we examined 72 patients with laryngeal squamous cell cancer (LSCC), the most common type of HNSCC, in this study (Table 1). Double immunohistochemical staining revealed substantial infiltration of aTreg cells in the peritumoral area and stroma of tumors (Figure 1E). All tumor-infiltrating FoxP3^+^ cells were CD25^+^ T cells, while 93.6 ± 8.8% of CD25^+^ T cells were FoxP3^+^ cells in the tumor tissue. The median level of aTreg cell infiltration was 3.75 (range: 0–24) in the whole population. When the median value was used as a cutoff to define low and high levels of aTreg cell infiltration, the percentage of tumor differentiation was indicated (Figure 1F). We did not find a correlation between the infiltration level of aTreg cells and pathological stage (Figure 1G). However, the level of aTreg cell infiltration in patients at early clinical stages (I and II) was lower than that at late clinical stages (III and IV) (*P* < 0.001) (Figure 1H, 1I) (Supplementary Table 1).

**Table 1:** Clinicopathological features of LSCC patients

		No. cases	%
Gender	Male	68	94.40
	Female	4	5.60
Age (year)	< 61	35	48.61
	≥ 61	37	51.39
Tumor grade	G_1_	34	47.22
	G_2_	27	37.50
	G_3_	11	15.28
Tumor status	T_1–2_	42	58.33
	T_3–4_	30	41.67
Nodal status	N_0_	58	80.56
	N_1–2_	14	19.44
Stage (UICC 2008)	I–II	38	52.78
	III–IV	34	47.22
M stage	M_0_	72	100.00
	M_1_	0	0.00

LSCC: Laryngeal squamous cell carcinoma.

We hypothesized that tumor-infiltrating aTreg cells would adversely correlate with survival. In univariate analysis, the low level group was associated with a longer survival time (*p* = 0.001) (Figure 1J). Survival was still significantly different for the group at stages III and IV (*p* = 0.036; median: 9.75) (Figure 1K), but not stages I and II (*P* = 0.49; median: 2.50) (Figure 1L). Therefore, an increase in the number of tumor-infiltrating aTreg cells was a significant predictor of reduced survival in patients with LSCC.

In a Cox multivariate analysis, only two variables influenced the overall survival probability: clinical stage (*p* = 0.04; relative risk: 1.65) and the level of infiltration of aTreg cells (*p* = 0.035; relative risk: 4.05; Supplementary Table 2). Differences in treatment modalities and other factors known to correlate with survival were included in this model and did not change the significance of these variables.

### aTreg cells suppress TAA immunity *in vitro*

**Figure 2:**
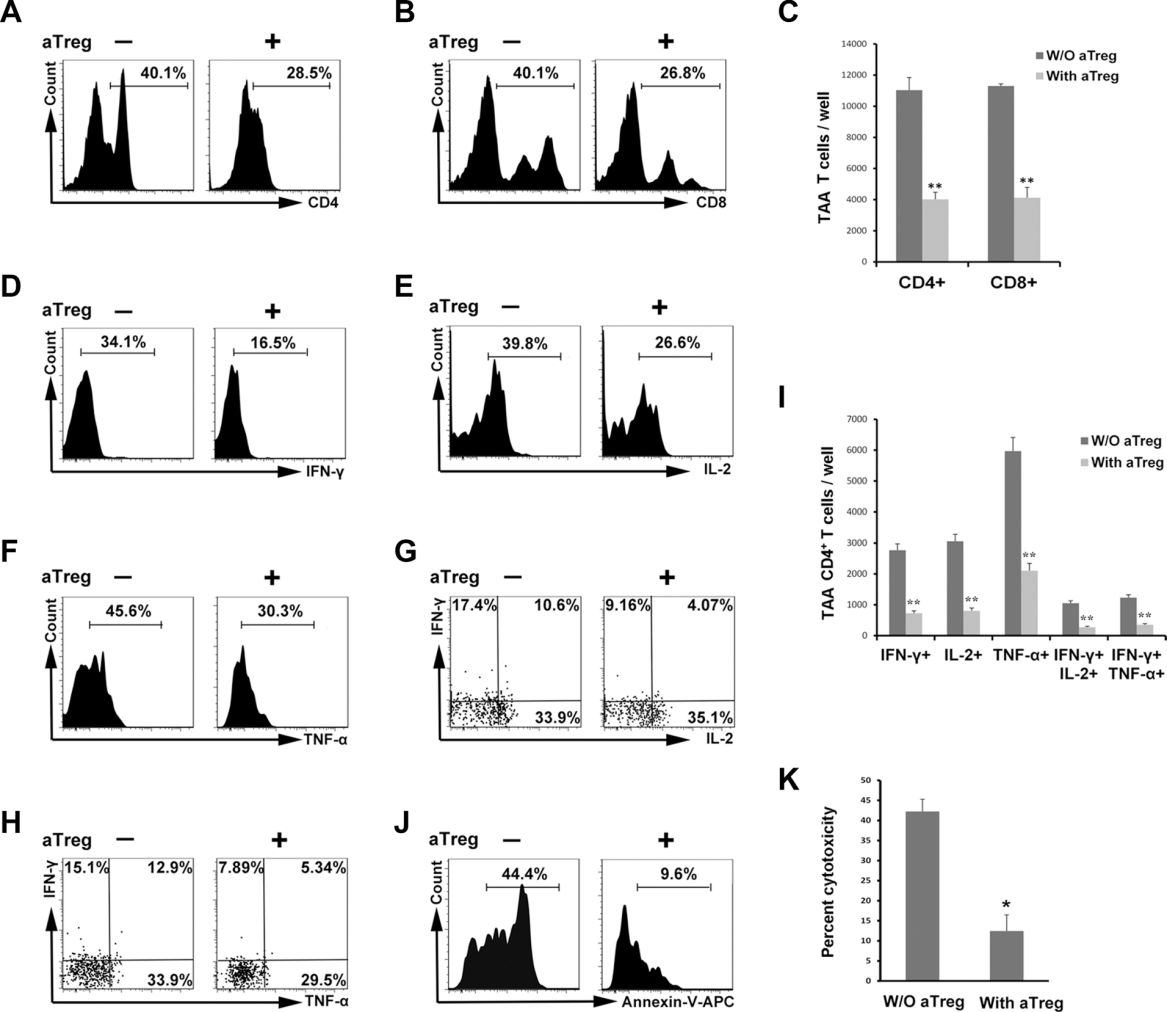
aTreg cells inhibit TAA immunity *in vitro* (**A–C**) Inhibition of TAA T cell proliferation by aTreg cells. Antigen-loaded mature DCs (TAA-mDCs) were added to autologous T cells in the presence of aTreg cells. Six days after the second stimulation, (**A**) CD4^+^ and (B) CD8^+^ T cell proliferation were assessed by flow cytometry. (C) Statistical comparisons were performed using Student's *t*-tests. **(D–I**) Inhibition of TAA CD4^+^ T cell production of IFN-γ, IL-2, and TNF-α by aTreg cells. T cells were stimulated with TAA-mDCs, and Th1 cytokines detected by intracellular staining. Secretion of (D) IFN-γ, E. IL-2, and (F) TNF-α, and the frequencies of Th1 subsets ((G) IFN-γ^+^IL-2^+^ and (H) IFN-γ^+^ TNF-α^+^) were significantly inhibited by aTreg cells. (I) Statistical comparisons were performed using Student's *t*-tests. (**J–K**) Inhibition of TAA T cell cytotoxicity by aTreg cells. (**J**) The cytotoxicity of TAA-immature DCs was analyzed by flow cytometry with annexin V and 7-AAD staining. (K) Experiments were performed in triplicate and repeated twice. A representative experiment is shown. Statistical comparisons were performed using Student's *t*-tests. *P < 0.05 and **P < 0.01.

To evaluate the suppressive functions of aTreg cells in TAA immunity, immature DCs were loaded with soluble antigens from a cancer cell lysate (SNU899 laryngeal cancer cell line), and then co-cultured with autologous lymphocytes, which induced specific T cell activation as expected (Supplementary Figure 1). This induction of TAA CD4^+^ and CD8^+^ T cell proliferation was markedly inhibited by allogeneic aTreg cells that were prepared as described previously [24, 25] (Figure 2A–2C). Moreover, the frequencies of interferon (IFN)-γ^+^, IL-2^+^ and tumor necrosis factor (TNF)-α^+^CD4^+^ T cells were reduced by aTreg cells (Figure 2D–2F). The Th1 subset (IFN-γ^+^IL-2^+^ and IFN-γ^+^TNF-α^+^) decreased by more than 50% when aTreg cells were added to the co-culture system (Figure 2G, 2H). We quantified the number of cytokine-secreting cells per well in 96-well plates and determined that the numbers of IFN-γ^+^, IL-2^+^, TNF-α^+^, IFN-γ^+^IL-2^+^, and IFN-γ^+^TNF-α^+^ cells were significantly reduced in the presence of aTreg cells (Figure 2I, *P* < 0.01 for all).

Finally, the cytotoxicity of antigen-loaded autologous immature DCs was assessed because the major histocompatibility complex (MHC) could be matched to reactive cytotoxic T lymphocytes [27]. The addition of aTreg cells resulted in a decrease in the average percentage of cytotoxicity from 42.3% to 12.5% (Figure 2J, 2K, *P* < 0.05), indicating that aTreg cells blocked the protective effects of T cells in the tumor. These data indicated that aTreg cells suppressed TAA effector T cell immunity in patients with HNSCC.

### CCR4 is predominantly expressed on aTreg cells

**Figure 3:**
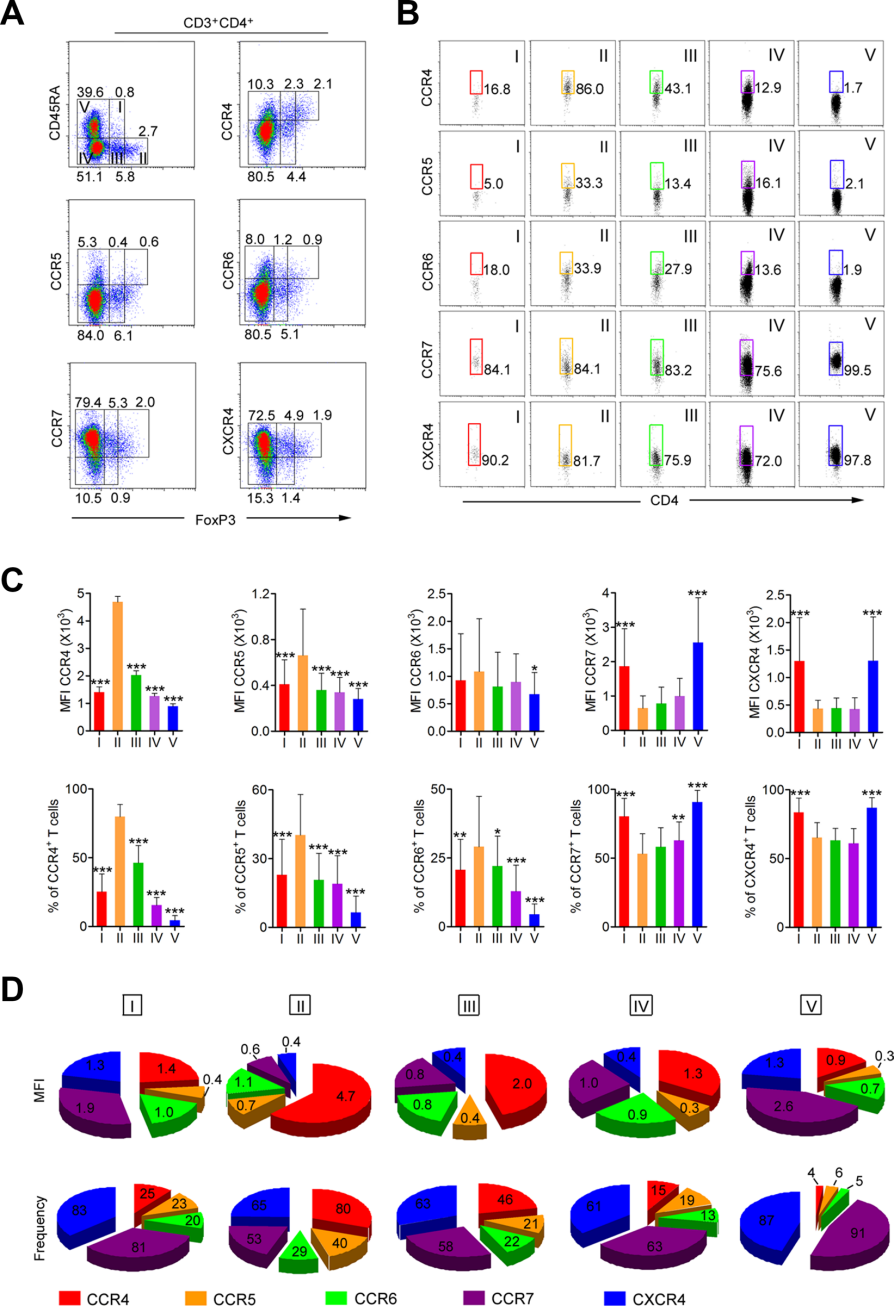
CCR4 is predominantly expressed on aTreg cells in HNSCC patients (**A**) CCR4, CCR5, CCR6, CCR7, and CXCR4 expression by subpopulation of FoxP3^+^ Treg cells in PBMCs from HNSCC patients. (**B**) The expression of CCR4, CCR5, CCR6, CCR7, and CXCR4 was evaluated in each fraction of CD4^+^ T cells. Representative data from 50 HNSCC patients are shown. (**C**) Mean fluorescence intensity (MFI, upper) and frequency (lower) of CCR4, CCR5, CCR6, CCR7, and CXCR4 expression by each fraction of CD4^+^ T cells in PBMCs from HNSCC patients (*n* = 50). Statistical differences were analyzed using Kruskal-Wallis tests. *P < 0.05, **P < 0.001, and ***P < 0.0001. (**D**) Summary of MFIs (mean, upper) and frequency (mean, lower) of CCR4, CCR5, CCR6, CCR7, and CXCR4 in each fraction of CD4^+^ T cells. I: FoxP3^lo^CD45RA^+^CD4^+^ resting Treg cells; II: FoxP3^hi^CD45RA^−^CD4^+^ aTreg cells; III: cytokine-secreting FoxP3^lo^CD45RA^−^CD4^+^ non-Treg cells; IV: FoxP3^−^CD45RA^−^CD4^+^ T cells; V: FoxP3^−^CD45RA^+^CD4^+^ T cells.

To identify proteins involved in the recruitment of circulating aTreg cell to HNSCC tumors, we compared the expression of CCR4, CCR5, CCR6, CCR7, and C-X-C chemokine receptor (CXCR) 4 [3, 7, 26] in circulating FoxP3^+^CD25^+^CD4^+^ Treg cells from HNSCC patients (Supplementary Figure 2). We then focused on the expression of these chemokine receptors on FoxP3^+^CD25^+^CD4^+^ T cell subsets and FoxP3^−^CD4^+^ T cells. The results showed that chemokine receptor-positive T cells were present in both the FoxP3^+^ and FoxP3^−^ T cell fractions (Figure 3A). When FoxP3^+^ T cells were classified into three subsets according to FoxP3 and CD45RA expression [24, 25], only FoxP3^hi^CD45RA^−^aTreg cells (Fr. II) predominantly expressed CCR4; FoxP3^lo^CD45RA^+^ rTreg cells (Fr. I) exhibited low CCR4 expression and FoxP3^lo^CD45RA^−^ non-Treg cells (Fr. III) exhibited moderate expression. Among the FoxP3^−^ cells, some CD45RA^−^CD4^+^ memory and activated T cells (Fr. IV) expressed CCR4, while CD45RA^+^CD4^+^ naive T cells (Fr. V) did not (Figure 3B). Analysis of the expression of four other chemokine receptors (CCR5, CCR6, CCR7, and CXCR4) revealed that the expression of these chemokine receptors on the above CD4^+^ T cell fractions did not show the same pattern as CCR4. Specifically, aTreg cells (Fr. II) and the four other fractions (Fr. I, III, IV, and V) exhibited low CCR5 and CCR6 expression (Figure 3B). Although high expression of CCR7 and CXCR4 was observed in aTreg cells, the other four fractions exhibited high expression (Figure 3B), indicating that CCR7 and CXCR4 could not be used for selective blockade of aTreg cell trafficking in tumors. Mean fluorescence intensity (MFI) and frequency analysis of multiple samples of peripheral blood mononuclear cells (PBMCs) from HNSCC patients showed similar patterns of chemokine receptor expression in FoxP3^+^ subsets (Figure 3C, 3D). Moreover, PBMCs from healthy individuals showed similar patterns of CCR4 expression in FoxP3^+^ subsets (Supplementary Figure 3).

To confirm that CCR4 blockade could be used to selectively prevent circulating aTreg cell trafficking, we analyzed the expression of CCR4 on various types of immune cells. The results indicated that CD8^+^ T cells, activated CD4^+^ T cells, natural killer (NK) cells, CD14^+^ monocytes/macrophages, dendritic cells (DCs), and B cells did not significantly express CCR4 (Supplementary Figure 4). Finally, we compared the expression of CCR4 on HNSCC tumor-infiltrating aTreg cells and matched circulating aTreg cells from 18 HNSCC patients. These results demonstrated that tumor-infiltrating aTreg cells predominantly expressed CCR4, which was consistent with the expression pattern in circulating aTreg cells (Supplementary Figure 5). Thus, inhibition of CCR4 blocked aTreg cell trafficking in HNSCC.

### MCP-1 is highly expressed in the HNSCC microenvironment

**Figure 4:**
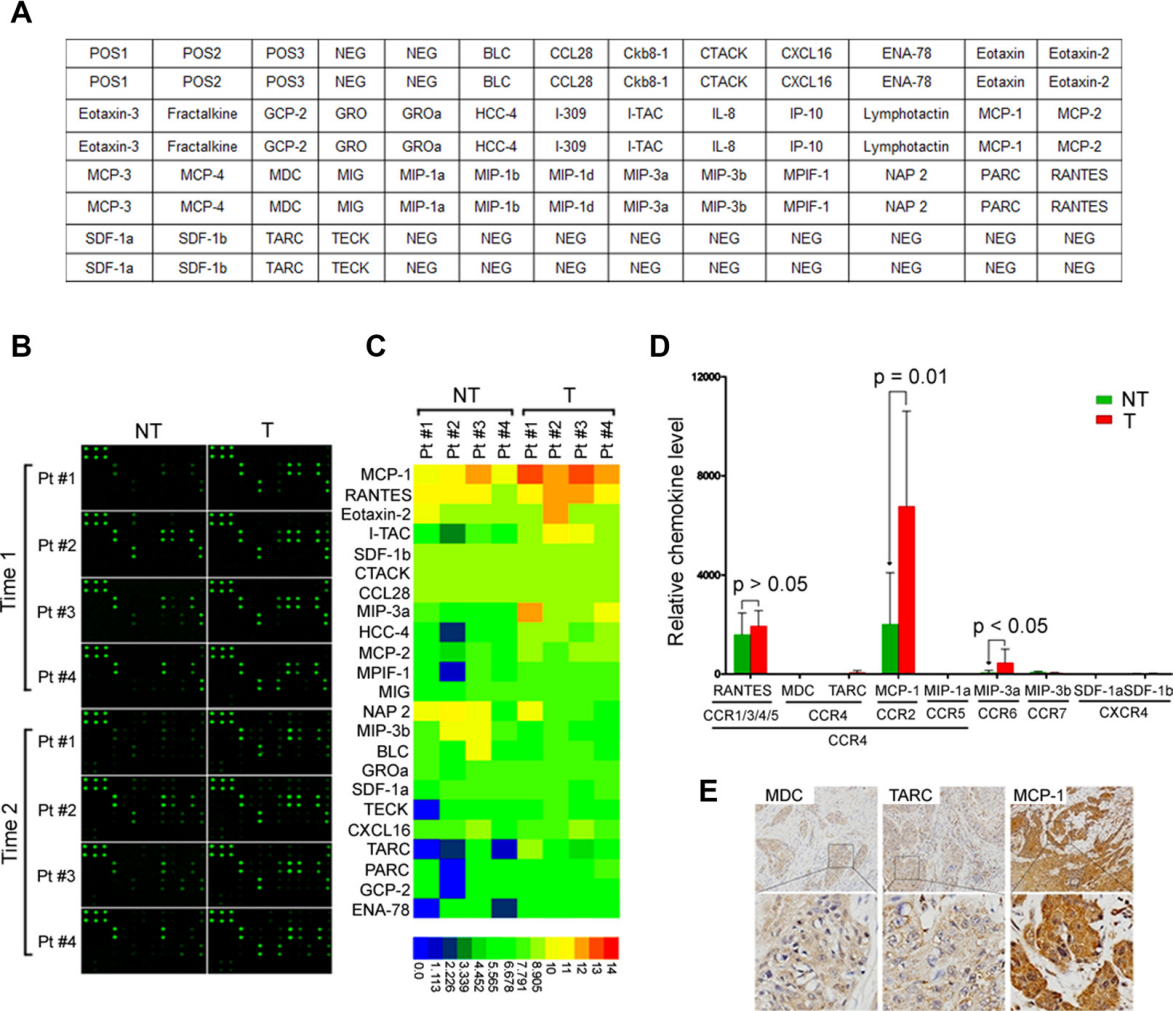
MCP-1 is increased in tumor tissues from HNSCC patients (**A**) Map of antibodies against chemokines on the RayBiotech Human Chemokine Antibody Array (AAH-CHE-G1). (**B**) Cell lysates from tumor and adjacent nontumor tissues were first applied to the array. Bound chemokines were then recognized by a pool of anti-chemokine antibodies corresponding to the antibodies spotted on the array. Similar results were observed in two independent arrays. (**C**) Semiquantification of scanned antibody arrays. The levels were normalized to internal positive controls present in each membrane. Semiquantitative levels are represented in the heat map. (**D**) Expression of CCR4, CCR5, CCR6, CCR7, CXCR4 ligands in tumor and adjacent nontumor tissues. Statistical differences were analyzed using Mann-Whitney *U*-tests. (**E**) Representative staining of HNSCC cases for MDC, TARC, and MCP-1 (*n* = 15). Brown: positive staining × 100. Lower panels are magnified images of the boxed area in the corresponding upper panel. NT: nontumor; T: tumor.

CCR4 is a receptor for monocyte chemoattractant protein-1 (MCP-1), regulated on activation, normal T cell expressed and secreted (RANTES), macrophage inflammatory protein-1a (MIP-1a), thymus and activation regulated chemokine (TARC), and macrophage-derived chemokine (MDC). We investigated whether the expression one of these endogenous CCR4-binding ligands was specifically increased in the HNSCC microenvironment. We used antibody arrays (RayBiotech) to screen the levels of 38 chemoattractant factors (Figure 4A, 4B) in paired tumor and adjacent nontumor tissues from four HNSCC patients. Approximately 23 secreted factors were detected in both tumor tissues and adjacent nontumor tissues (Figure 4C). Interestingly, among the five chemoattractant ligands for CCR4, only MCP-1 was increased in tumor tissues compared with adjacent nontumor tissues, while RANTES, MIP-1a, TARC, and MDC were not. The expression of CCR5, CCR6, CCR7, and CXCR4 ligands was low or undetectable (Figure 4D). To confirm endogenous MCP-1 expression within the HNSCC microenvironment, tissue sections from 15 HNSCC biopsies were stained using antibodies specific for MCP-1 and two unique chemoattractant ligands (MDC and TARC) for CCR4 (Figure 4E). The immunohistochemistry data were consistent with that of the antibody array. Therefore, we postulated that HNSCC-circulating aTreg cells could be recruited to the tumor microenvironment by endogenous MCP-1 through binding to CCR4.

### MCP-1/CCR4 signaling increases circulating aTreg cell trafficking in tumors

**Figure 5:**
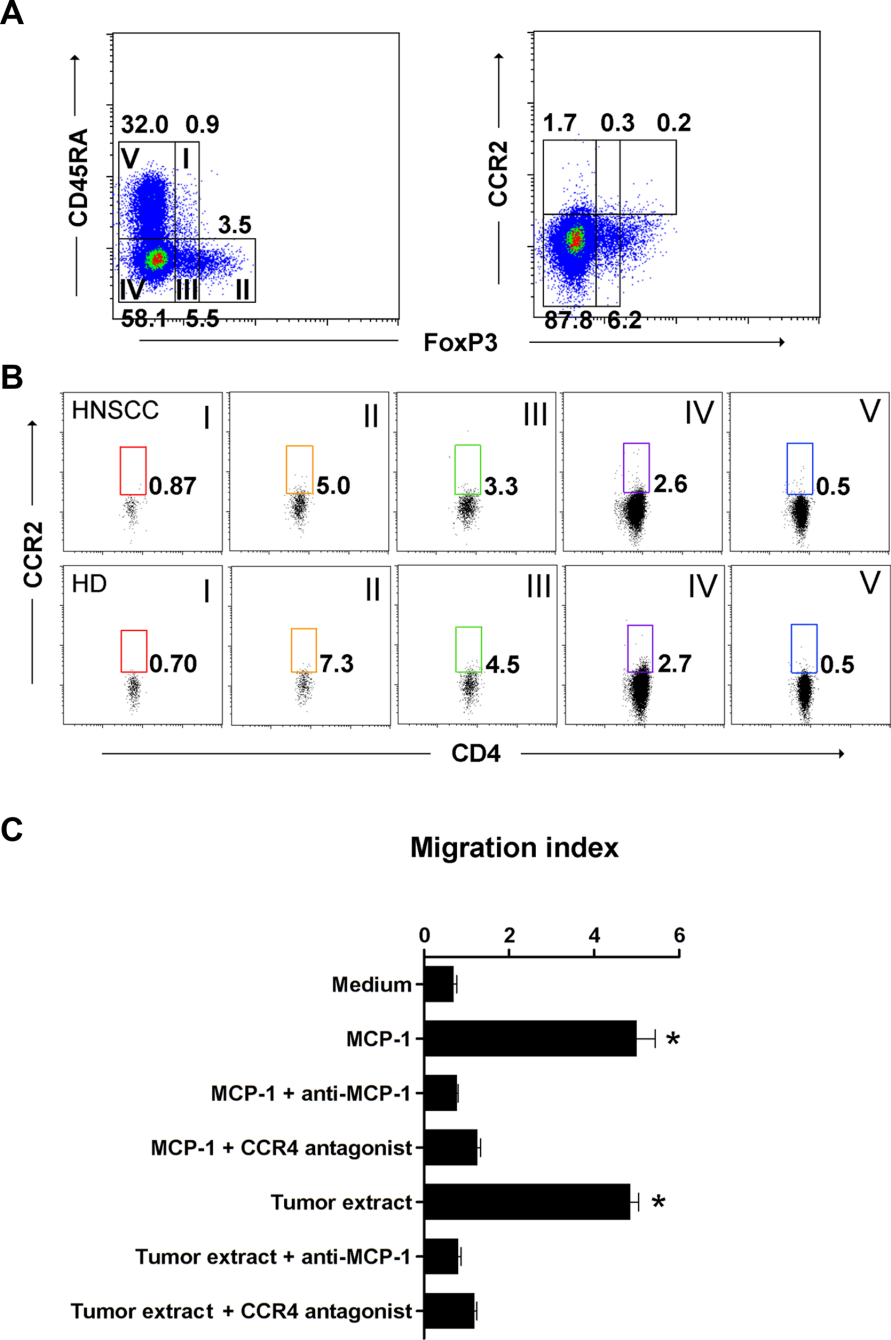
MCP-1/CCR4 signaling promotes aTreg cell migration (**A**), (**B**) CCR2-positive T cells were scarcely present in fractions of FoxP3^+^CD4^+^ and FoxP3^−^CD4^+^ T cells. (**A**) CCR2 expression by subpopulation of FoxP3^+^ Treg cells in PBMCs from HNSCC patients and healthy donors. (B) Expression of CCR2 in CD4^+^ T cell fractions. Representative data from 19 HNSCC patients and 12 healthy donors are shown. (**C**) Migration of aTreg cells in response to a tumor extract or recombinant MCP-1. A specific anti-MCP-1 antibody or CCR4 antagonist significantly inhibited aTreg cell migration. Results are the mean ± SD. Statistical differences were analyzed by one-way analysis of variance (ANOVA). *P < 0.01.

MCP-1 is a chemoattractant ligand for both CCR4 and CCR2. Therefore, we could not conclude that CCR4 was the only functional receptor in MCP-1-induced circulating aTreg cell recruitment. To elucidate the specific chemoattractant responsible for aTreg cell recruitment, we first analyzed CCR2 expression on circulating aTreg cells from both HNSCC patients and healthy donors. CCR2^+^ T cells were nearly absent in FoxP3^+^CD4^+^ T cell fractions, indicating that HNSCC-circulating aTreg cells may be recruited to the tumor microenvironment by endogenous MCP-1 via binding to CCR4 but not CCR2 (Figure 5A, 5B).

We next examined whether the CCR4 on aTreg cells was functional. Freshly isolated circulating aTreg cells from HNSCC patients were analyzed in migration assays (*n* = 3). The data showed that freshly isolated tumor extracts induced significant migration of aTreg cells *in vitro* (*P* < 0.01 versus control [media]). In addition, both a monoclonal antibody against human MCP-1 and a CCR4 antagonist significantly blocked tumor extract-induced aTreg cell migration (*P* < 0.01 for each). In support of these results, recombinant MCP-1 induced significant aTreg cell migration (*P* < 0.01 versus control) that was efficiently blocked by the anti-MCP-1 antibody and CCR4 antagonist (*P* < 0.01 for each) (Figure 5C). Thus, MCP-1 could increase aTreg cell trafficking via binding to CCR4 *in vitro* and could recruit aTreg cells to tumors.

### A CCR4 antagonist evokes and enhances antitumor immune responses *in vivo*

**Figure 6:**
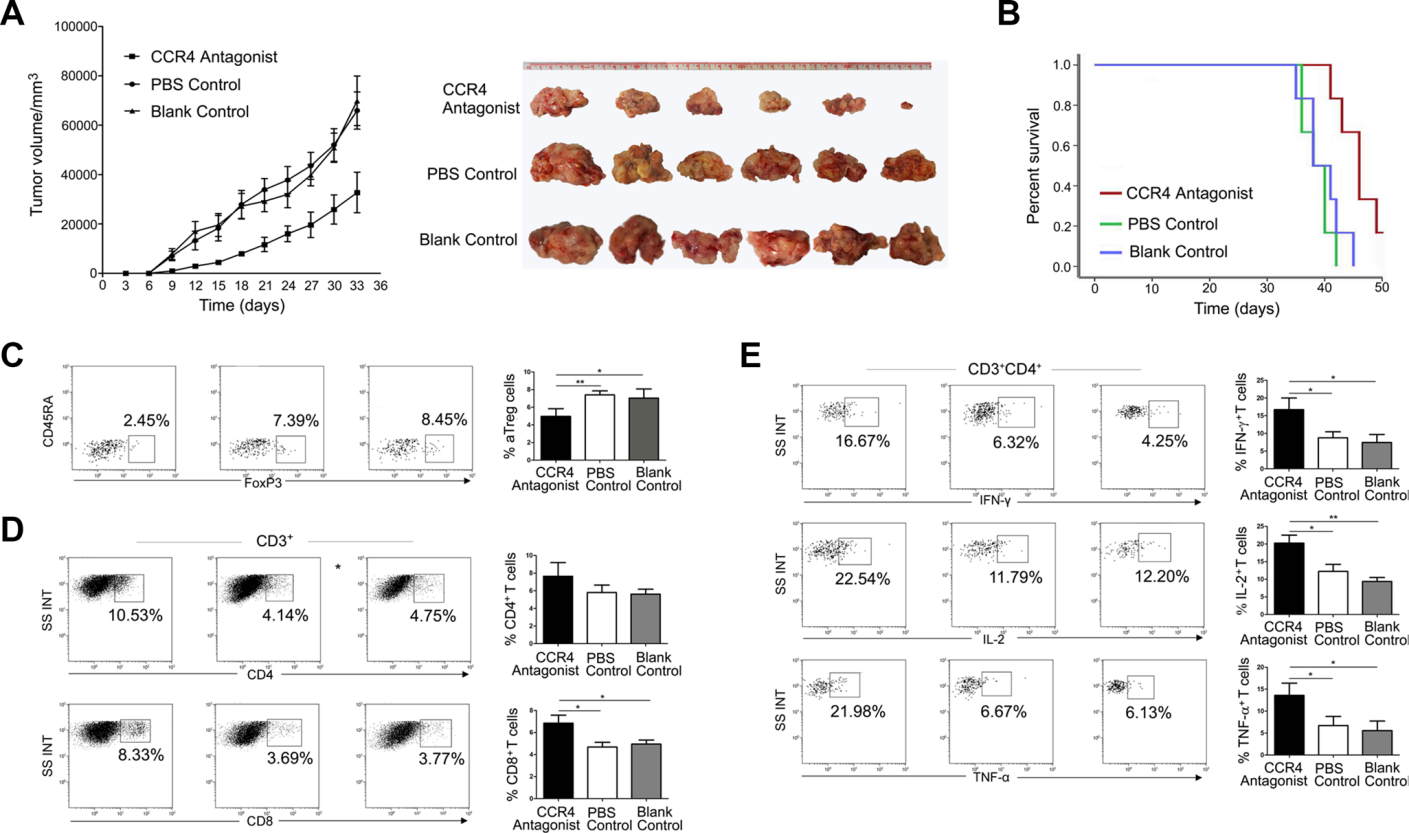
A CCR4 antagonist blocks aTreg cell trafficking in tumors leading to enhanced antitumor immune responses and prolonged survival (**A**) Tumor sizes were measured every 3 days after tumor cell injection (left panel). Tumors isolated 33 days after injection are shown in the right panel. Data are presented as the mean ± SD. *P* < 0.05 for the PBS or blank control groups versus the CCR4 antagonist-treated group. (**B**) Survival depicted as the percentage of surviving mice on the indicated day after tumor cell injection. Statistical comparisons were performed using the Kaplan-Meier test. *P* < 0.05 for the PBS or blank control groups versus the CCR4 antagonist-treated group. Frequencies of (**C**) aTreg cells, (**D**) CD4^+^, CD8^+^T cells, and E. CD4^+^ T cells that secreted IFN-γ, IL-2, and TNF-α. Histograms show the frequencies of each T cell subset. Statistical comparisons were performed using one-way ANOVA. *P < 0.05 and **P < 0.01.

We next determined whether CCR4-binding treatment could evoke and enhance antitumor immunity *in vivo* (*n* = 6 per group, C3H-HeN mice). After administration of a CCR4 antagonist, we observed inhibition of SCC-VII tumor growth at day 33 (*P* < 0.01 for each, Figure 6A) and prolongation of the overall survival time (*P* < 0.05 for each, Figure 6B) in the CCR4 antagonist-treated group compared to the control groups. One mouse in the CCR4 antagonist-treated group was still alive at the end of the observation period at day 50. The frequency of tumor-infiltrating aTreg cells was also lower in the CCR4 antagonist-treated group than in the PBS/blank control groups (*P* < 0.05 for each), suggesting that the CCR4 antagonist inhibited aTreg cell migration *in vivo* (Figure 6C). The frequency of tumor-infiltrating CD4^+^ T cells was higher in the CCR4 antagonist-treated group than in the control groups, but the difference was not statistically significant. The frequency of tumor-infiltrating CD8^+^ T cells in the CCR4 antagonist-treated group was higher than in the control groups (*P* < 0.05 for each, Figure 6D). We next examined the cytokine profiles of tumor-infiltrating lymphocytes following *ex vivo* stimulation, and found that T cells in the CCR4 antagonist-treated group secreted significantly higher amounts of IFN-γ, IL-2, and TNF-α than T cells in the control groups (*P* < 0.05 for each) (Figure 6E).

## DISCUSSION

Accumulating evidence indicates that FoxP3^+^ Treg cells can suppress tumor-specific immunity, which provides a clear rationale for development of immunotherapies that can modulate the influence of these regulatory cells [3, 17, 28]. Here, we investigated the distribution of FoxP3^+^ T cell subsets in HNSCC tissues. Intriguingly, our data showed that the majority of tumor-infiltrating FoxP3^+^ T cells were aTreg cells. Although there is a large amount of evidence that FoxP3^+^ T cells predominantly infiltrate tumor tissues [6, 7, 17, 29, 30], the detailed phenotypes of these FoxP3^+^ T cells have not been characterized. Our finding of predominant aTreg cell infiltration revealed the phenotypes of such cells. Additionally, aTreg cell numbers were significantly higher in late-stage (III and IV) HNSCC tumor tissue than in early-stage tissue (I and II), and were inversely correlated with patient prognosis. Given the clinical importance of tumor-infiltrating aTreg cells, inhibition of aTreg cell recruitment in HNSCC may be an effective immunotherapeutic strategy.

We recently confirmed that aTreg cells suppress proliferation of non-TAA T cells [24, 25]. However, there has been no definitive demonstration of the role of aTreg cells in TAA immunity. Our data showed that aTreg cells from HNSCC patients inhibit TAA immunity, which resulted in lower TAA-loaded cell cytotoxicity *in vitro*. These findings provide a rationale for the development of immunotherapies to control the effects of aTreg cells in HNSCC.

Since chemokine receptors have central roles in the recruitment of immunoregulatory cells [7, 17, 31, 32, 33], we focused on the expression of these receptors in aTreg cells in order to identify a specific chemokine receptor that could be inhibited to block aTreg cell trafficking. The data indicated that aTreg cells predominantly expressed CCR4 compared with FoxP3^+/−^CD4^+^ T cell subsets and other types of immune cells. This result was consistent with a previous study, which showed that CCR4 was predominantly expressed in peripheral blood aTreg cells from melanoma patients [34]. These data strongly suggest that blockade of aTreg cells by therapeutics that bind CCR4 may be used to selectively prevent circulating aTreg cell trafficking.

Circulating FoxP3^+^ Treg cells express CCR4 and migrate in response to TARC and MDC [35–38]. However, it is unknown whether these chemoattractants are involved in aTreg cell homing to HNSCC tumors. Here, we showed that TARC and MDC were not significantly expressed in HNSCC tissues. In contrast, MCP-1, a chemoattractant ligand for both CCR2 and CCR4, was highly expressed in HNSCC tissues compared to adjacent nontumor tissues. This finding indicated that MCP-1, but not TARC or MDC, was involved in aTreg cell trafficking in tumors via CCR2 and/or CCR4. CCR2 is expressed on blood lymphocytes such as monocytes, NK cells, and B cells [39–41]. However, whether aTreg cells expressed CCR2 was unclear. We observed low expression of CCR2 on aTreg cells. In addition, we determined that circulating aTreg cells, which highly express functional CCR4, migrated toward MCP-1 in the tumor microenvironment.

Our *in vivo* studies showed that inhibition of tumor growth and prolonged mouse survival could be attributed to inhibition of aTreg cell recruitment resulting in expansion of tumor infiltrating CD4^+^ and CD8^+^ T cells. These data demonstrate that aTreg cells are involved in the progression of HNSCC via their suppressive effects on TAA immune responses. Therefore, therapeutics that bind aTreg cells could be an effective immunotherapy for HNSCC. Several recent clinical trials have shown that depletion of CCR4-expressing FoxP3^+^CD4^+^ Treg cells by anti-human CCR4 monoclonal antibody is a promising approach to augment antitumor immune responses in cancer patients [42, 43]. The above findings give us confidence that inhibiting CCR4-expressing aTreg cell recruitment may be an effective strategy in HNSCC immunotherapy.

In conclusion, our data suggest that a combination of a CCR4-binding therapeutic that can block aTreg cell trafficking in tumors, tumor antigen immunization, and monoclonal antibodies may enhance the clinical efficacy of immunotherapies for HNSCC.

## MATERIALS AND METHODS

### Donor samples

PBMCs were obtained from healthy donors and HNSCC patients who had not received any previous oncological treatments. Tumor- and nontumor-infiltrating lymphocytes were obtained from tumor and adjacent nontumor tissues after surgical debulking. Informed consent was obtained from all participants prior to enrollment in the study. The study was approved by the Ethics Committee of The First Affiliated Hospital of Sun Yat-sen University, Guangzhou, China (Approval No. 2012-349).

### Preparation of infiltrating lymphocytes

Infiltrating lymphocyte suspensions were prepared by enzymatic digestion [44]. Dissected tumor or adjacent nontumor tissues were minced into small pieces with a scalpel. Samples (0.25 g) were then immersed in 10 mL of a digestion mixture [RPMI 1640 containing 5% fetal bovine serum, 0.5 mg/mL collagenase A, 0.2 mg/mL hyaluronidase type V, and 0.02 mg/mL DNase I (Sigma-Aldrich St. Louis, MO, USA)]. The samples were incubated on a rotating platform at 37°C for 30 min and the resulting cell suspensions filtered through 40-mm cell strainers (BD Biosciences, San Diego, CA, USA). Finally, lymphocyte suspensions were obtained using Ficoll-Hypaque (Haoyang Bio, Tianjing, China).

### Cell lines and mice

The SNU899 LSCC cell line was provided by Professor Ja-Lok Ku (Seoul National University College of Medicine, South Korea). The mouse SCC VII OSCC cell line was provided by Professor Si-Xi Liu (West China Hospital, China). Cells were cultured under standard conditions.

Eight-week old male C3H-HeN mice were purchased from Slac Laboratory Animal Co., Ltd. (Shanghai, China). All mice were housed under specific pathogen-free conditions. Experimental procedures were approved by the Institutional Animal Studies Committee and conducted in accordance with Institutional Animal Care and Use Committee guidelines.

### Preparation of DCs and TAA loading

CD14^+^ cells were isolated from PBMCs using CD14 Microbeads (Miltenyi Biotec, Bergisch Gladbach, Germany). The cells were then seeded into a 6-well plate at a density of 1.5 × 10^6^ cells/well and cultured as described previously [45]. On day 6, soluble SNU899 lysate antigens prepared by four freeze-thaw cycles (−140°C/42°C/60°C) were added to DC cultures at a ratio of 3:1 (SNU899 cells: DCs). On day 7, maturation of DCs was induced by the addition of 1 μg/mL lipopolysaccharide (Sigma-Aldrich) and the cells cultured for an additional 2 days. On day 9, DC phenotype was evaluated by flow cytometry (Gallios Flow Cytometer, Beckman Coulter, Hercules, CA, USA) using anti-hCD1a-PE-Cy7, anti-hCD83-FITC, anti-hHLA-DR-eFluor 450, anti-hCD80-PE-Cy5, anti-hCD86-PE, and anti-hCD40-APC (eBioscience, San Diego, CA, USA) antibodies.

### Intracellular cytokine secretion assay

After stimulation for 5 h with a cocktail of phorbol 12-myristate 13-acetate, ionomycin, and Golgi stop (brefeldin A and monensin) (eBioscience), cells were stained for cell surface markers and intracellular cytokines (IL-2, TNF-α, and IFN-γ) using anti-hTNF-α-Alexa Fluor 700, anti-hIL-2-PE-Cy7, and anti-hIFN-γ-APC-eFluor 780 antibodies as well as the Fixation/Permeabilization Buffer and Permeabilization Buffer from eBioscience.

### *In vitro* TAA immunosuppression in the presence of aTreg cells

Antigen-loaded DCs were added to autologous T cells as stimulators at a ratio of 1:20 in a round bottom 96-well microplate. The aTreg cells were prepared as described previously [24, 25] and added to cultures of antigen-loaded DCs and T cells at a ratio of 1:4. A second identical stimulation by antigen-loaded DCs was performed after 1 week. On day 13, CD4^+^ and CD8^+^ T cell proliferation and cytokine production of CD4^+^ T cells were assessed by flow cytometry using anti-hCD3-eFluor 450, anti-hCD4-FITC, anti-hCD8a-PE-Cy7, anti-hIFN-γ-APC-eFluor780, anti-hIL-2-PE-Cy7, and anti-hTNF-α-Alexa Fluor 700 antibodies (eBioscience). SNU899-derived, lysate-pulsed immature DCs were labeled with 5 μM 5,6-carboxyfluorescein diacetate succinimidyl ester (eBioscience) and mixed with T cells that had been cultured for 13 days at a ratio of 1:10 in 96-well microplates for 4 h in a 5% CO_2_ atmosphere at 37°C. Cytotoxicity was assessed by flow cytometry using APC-annexin V and 7-amino-actinomycin D (7-AAD) (BD Biosciences).

### Identification of FoxP3^+^CD4^+^ T cell subsets

To identify FoxP3^+^CD4^+^ T cell subsets, T cells obtained from tumor tissues, adjacent non-tumor tissues, and blood were stained with anti-hCD3-eFluor 605NC, anti-hCD4-FITC, anti-hCD25-APC, anti-hCD45RA-eFluor 450, and anti-hFoxP3-PE antibodies (eBioscience).

### Immunohistochemical analysis

The main clinical and pathological characteristics of LSCC patients are presented in Supplementary Table 4. Clinical staging and the anatomical site of the tumors were assessed according to the 6^th^ edition of the Union for International Cancer Control (UICC 2008) tumor-node-metastasis classification of malignant tumors. Detection of FoxP3 (Ab22510, IgG_1_, 1:50 dilution, Abcam, Cambridge, MA, USA) and CD25 (Ab61777, IgG, 1:100 dilution, Abcam) was performed on 4 μm-thick, paraffin-embedded sections of tissue samples with the indicated antibodies using a double staining kit (DS-0001, ZSGB-BIO, Beijing, China). For chemokine receptor ligand staining, sections of tissue samples were stained with anti-MCP-1, -MDC, or -TARC antibodies using an ABC kit (PK-4001, ZSGB-BIO).

### Chemokine receptor expression profiling

The expression of CCR2, CCR4, CCR5, CCR6, CCR7, and CXCR4 on lymphocytes was analyzed by flow cytometry using anti-hCD3-eFluor 605NC or anti-hCD3-eFluor 450, anti-hCD4-FITC, anti-hCD8-PE-Cy7, anti-hCD25-APC, anti-hCD45RA-eFluor 450, anti-hFoxP3-PE, anti-hCD14-FITC, anti-hCD19-PE-Cy7, anti-hCD11c-Alexa Fluor 700, anti-hCD16-PE, anti-hCD56-APC, anti-hCCR6-PerCP-eFluor710, anti-hCCR7-APC-eFluor780, and anti-hCXCR4-PE-Cy7 antibodies (eBioscience). The anti-hCCR2-Alexa Fluor 647, anti-hCCR4-PerCP-Cy5.5, and anti-hCCR5-APC-Cy7 antibodies were purchased from BD Pharmingen (San Diego, CA, USA).

### RayBiotech human chemokine antibody array

A Human Chemokine Antibody Array (RayBiotech, Inc, Norcross, GA, USA) was used according to the manufacturer's instructions. After development, films were scanned and the images processed and quantified using the National Institutes of Health ImageJ software. The intensities were normalized to internal positive controls for comparison.

### Migration assay

Migration was assessed as described previously [31] using 5 × 10^4^ aTreg cells. Human recombinant MCP-1 (0.5 μg/mL, R&D Systems, Minneapolis, MN, USA), a human anti-MCP-1 antibody (Monoclonal Mouse IgG1 Clone #24822, 15 μg/mL, R & D Systems), or tumor tissue extract were added to the lower chamber. The aTreg cells were preincubated with a CCR4 antagonist (C_27_H_41_N_5_O_2_.2HCl, 0.14 μM, TOCRIS, Minneapolis, MN, USA) for 30 min at 37°C. Migrated cells were counted using an automated cell counter (Scepter, Millipore, Billerica, MA, USA).

### *In vivo* augmentation of antitumor immune responses by the CCR4 antagonist

Eighteen C3H-HeN mice were randomly and equally divided into three groups and ear tagged prior to treatment. On day 0, 1 × 10^5^ tumor cells were injected subcutaneously into the right flank. Either a CCR4 antagonist (87.5 μg) or saline were injected intraperitoneally three times per week for 3 weeks from day 5 after tumor cell injection. In parallel, a group of six mice was used as a blank control. Tumor size was measured every 3 days using fine calipers. Tumor volumes were calculated as length × (width)^2^ × 0.52. All mice were sacrificed 7 days after the last injection.

After sacrifice, tumors were removed and single cell suspensions were prepared by enzymatic digestion. The types of tumor infiltrating cells were analyzed by flow cytometry using anti-mouse CD3-FITC, anti-mouse CD4-eFluor 450, anti-mouse CD8-Alexa Fluor 700, anti-mouse CD25-APC, anti-mouse IL-2-PE, anti-mouse IFN-γ-PE-Cy-5, anti-mouse TNF-α-PE-Cy7, anti-mouse FoxP3-PE, and anti-mouse CD45RA-FITC antibodies (Santa Cruz Biotechnology, Inc., Dallas, TX, USA), and a fixation/permeabilization kit. All antibodies were purchased from eBioscience unless otherwise specified. An additional three randomly divided groups of mice were used for survival analysis. The observation period was 50 days.

### Statistical analysis

Statistical analysis was performed with SPSS Standard version 13.0 software (IBM, Chicago, IL, USA). Differences between groups were assessed using Mann-Whitney *U*-tests, Student's *t*-tests, or Kruskal-Wallis tests. Comparisons of aTreg cell infiltration levels between groups were performed using the Wilcoxon matched-pairs signed-ranks test. Survival variables were estimated using the Kaplan-Meier method and compared using log-rank tests. Multivariate analysis using the Cox proportional hazard model was used to determine the influence of each variable, when adjusted to the others, on overall survival. A *P*-value of less than 0.05 was considered statistically significant.

## SUPPLEMENTARY MATERIALS FIGURES AND TABLES


