# Supplementary material for: Blockade of MCP-1/CCR4 signaling-induced recruitment of activated regulatory cells evokes an antitumor immune response in head and neck squamous cell carcinoma
Source: Oncotarget. 2016 May 10;7(25):37714–27. doi: 10.18632/oncotarget.9265 (PMC5122343; doi:10.18632/oncotarget.9265)
Supplement: Supplementary file 1 [file oncotarget-07-37714-s001.pdf]

Blockade of MCP-1/CCR4 signaling-induced recruitment of activated regulatory cells evokes an antitumor immune response in head and neck squamous cell carcinoma

Supplementary Materials

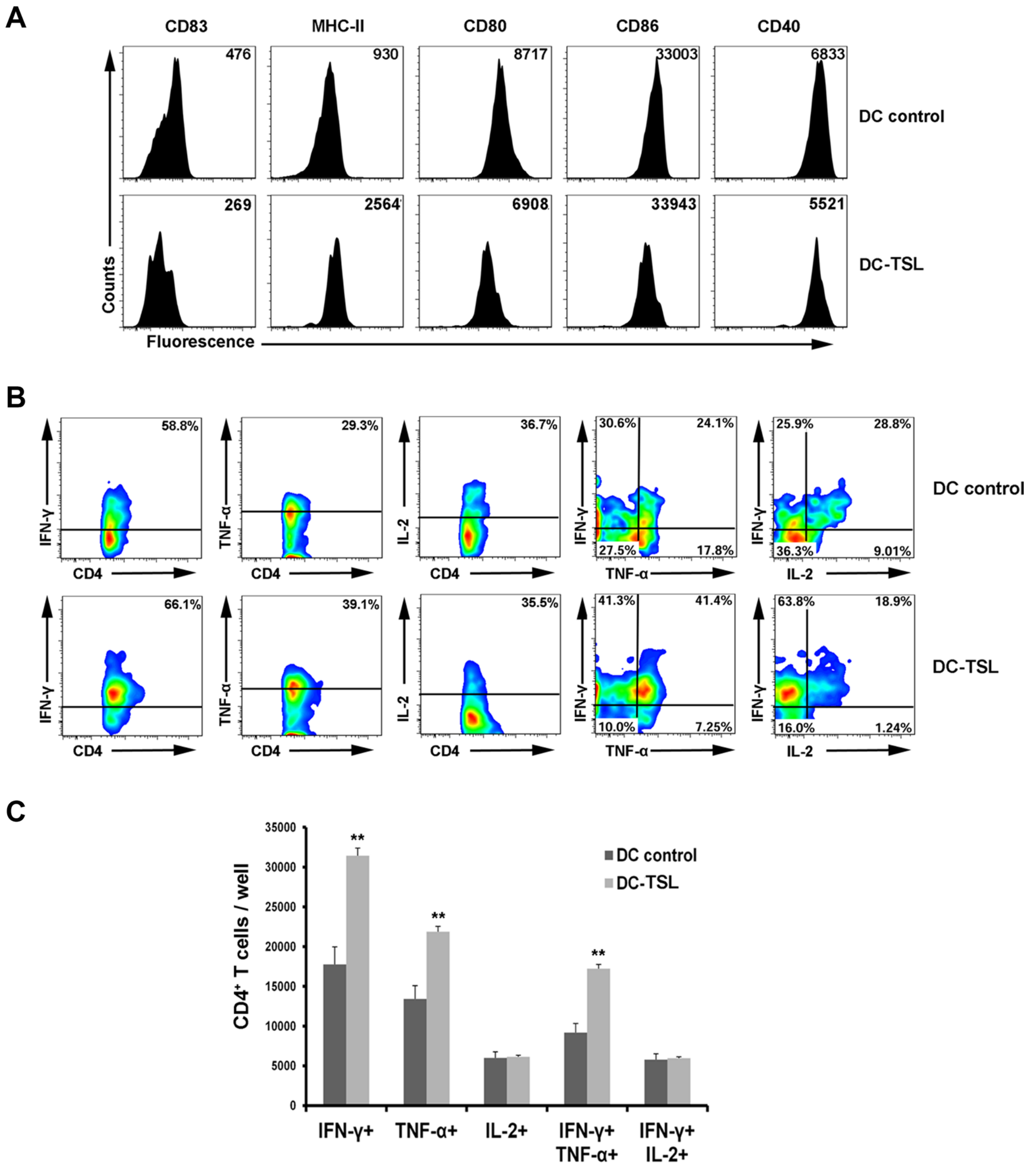

Supplementary Figure S1: Tumor lysate-exposed DCs stimulate cytokine production in autologous T cells. (**A**) Expression of DC markers, MHC, and co-stimulatory molecules on antigen-loaded DCs (DC-TSL) and a DC control. (**B**) The frequencies of IFN-γ^+CD4$^+$, TNF-α^+CD4$^+$, and IFN-γ^+TNF-α^+CD4$^+$ T cells were higher than in the control. (**C**) Statistical comparisons were performed using Student's *t*-tests. Data are the mean ± SD for triplicate experiments. $^{**}P < 0.01$. DC-TSL: DC pulsed with tumor-stressed lysates.

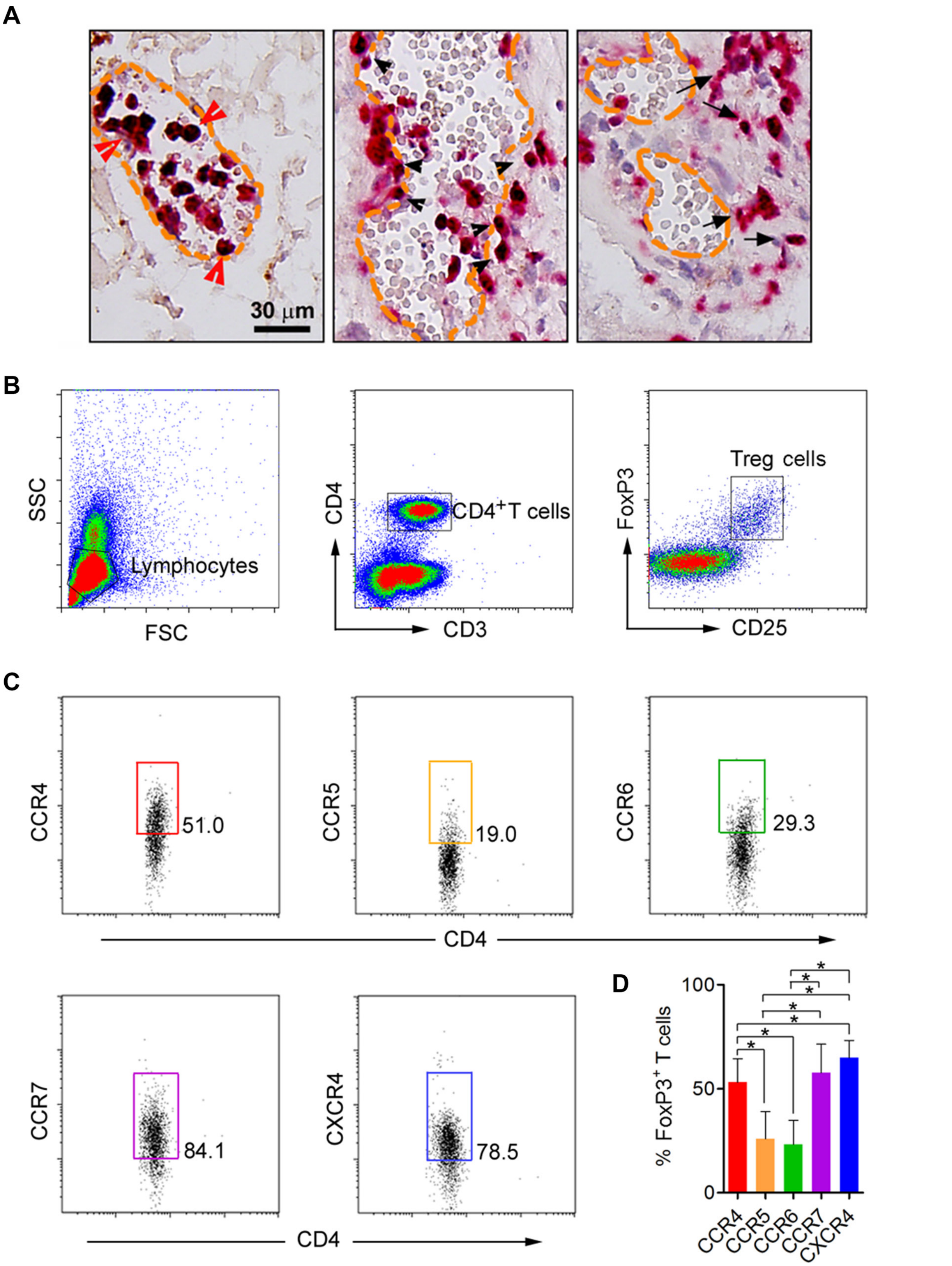

Supplementary Figure S2: Recruitment of aTreg cells and expression of chemokine receptors on circulating FoxP3$^+$CD25$^+$CD4$^+$ Treg cells from HNSCC patients. (**A**) Representative images of aTreg-cell recruitment from blood vessels to the tumor microenvironment (red arrowheads: aTreg cells in vessels; black arrowheads: aTreg cells adjacent to vessels; black arrows: aTreg cells distant from vessels). Brown: FoxP3; Red: CD25. Scale bars: 30 mm. (**B**) The gating strategy used to identify the frequency of FoxP3$^+$CD25$^+$CD4$^+$ Treg cells in the total CD3$^+$CD4$^+$ T cell population. (**C**) Representative images showing the expression of chemokine receptors CCR4, CCR5, CCR6, CCR7, and CXCR4 on circulating Treg cells. (**D**) Statistical differences between the five groups were analyzed using the Kruskal-Wallis test. *$P < 0.001$.

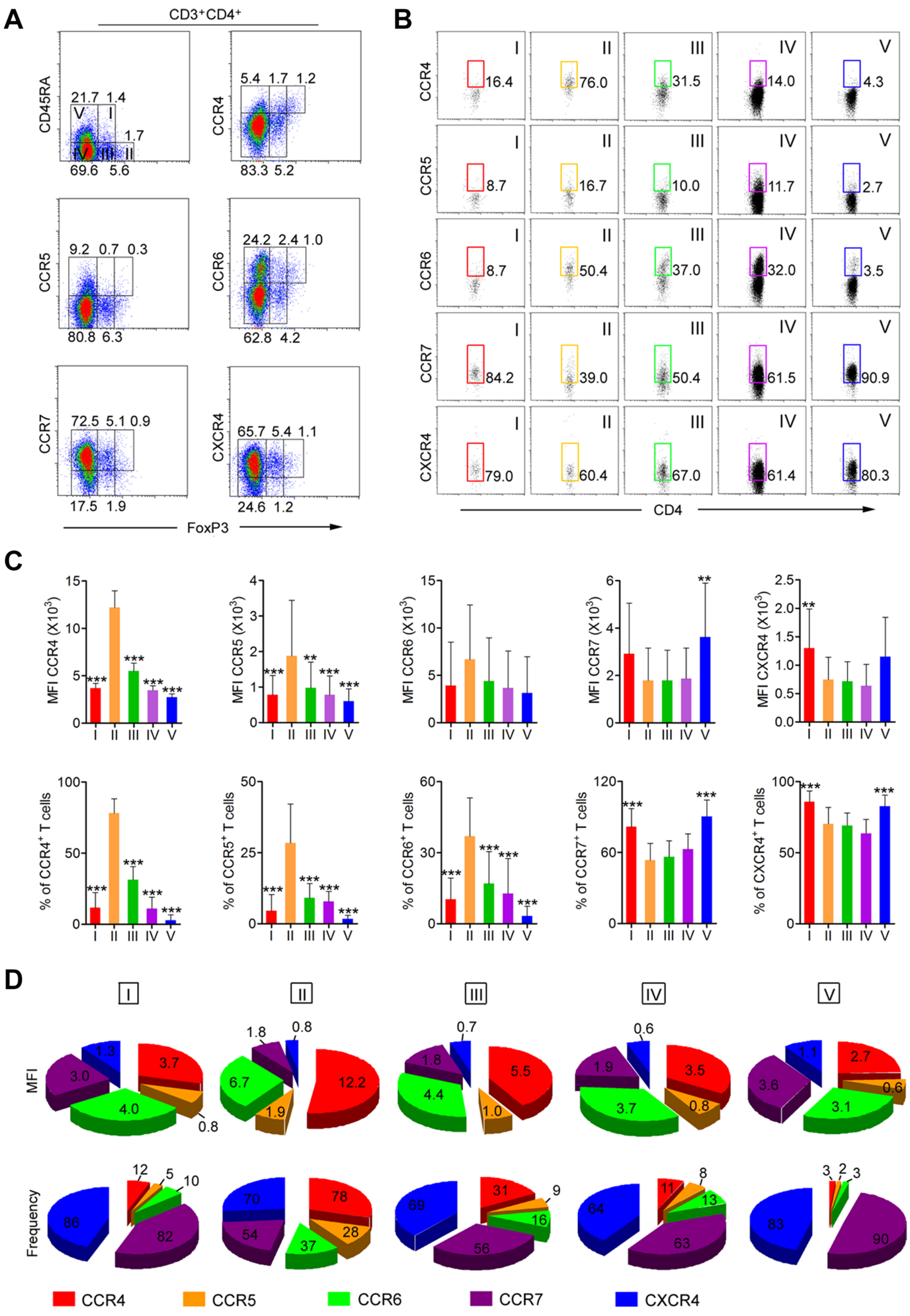

Supplementary Figure S3: CCR4 is predominantly expressed on aTreg cells from healthy donors. (**A**) CCR4, CCR5, CCR6, CCR7, and CXCR4 expression by subpopulation of FoxP3$^+$ Treg cells in PBMCs from healthy donors. (**B**) CCR4, CCR5, CCR6, CCR7, and CXCR4 expression were evaluated in each fraction of CD4$^+$ T cells. Representative data from 22 healthy donors are shown. (**C**) Mean fluorescence intensity (MFI, upper) and frequency (lower) of CCR4, CCR5, CCR6, CCR7, and CXCR4 expression by each fraction of CD4$^+$ T cells in PBMCs from healthy donors (n = 22). Statistical differences were analyzed using the Kruskal-Wallis test. *$P < 0.05$, **$P < 0.001$, and ***$P < 0.0001$. (**D**) Summary of MFIs (means, upper) and frequencies (means, lower) of CCR4, CCR5, CCR6, CCR7, and CXCR4 in each fraction of CD4$^+$ T cells. I: FoxP3^{lo}CD45RA$^+$CD4$^+$ resting Treg cells; II: FoxP3^{hi}CD45RA$^-$CD4$^+$ aTreg cells; III: cytokine-secreting FoxP3^{lo}CD45RA$^-$CD4$^+$ non-Treg cells; IV: FoxP3$^-$CD45RA$^-$CD4$^+$ T cells; V: FoxP3$^-$CD45RA$^+$CD4$^+$ T cells.

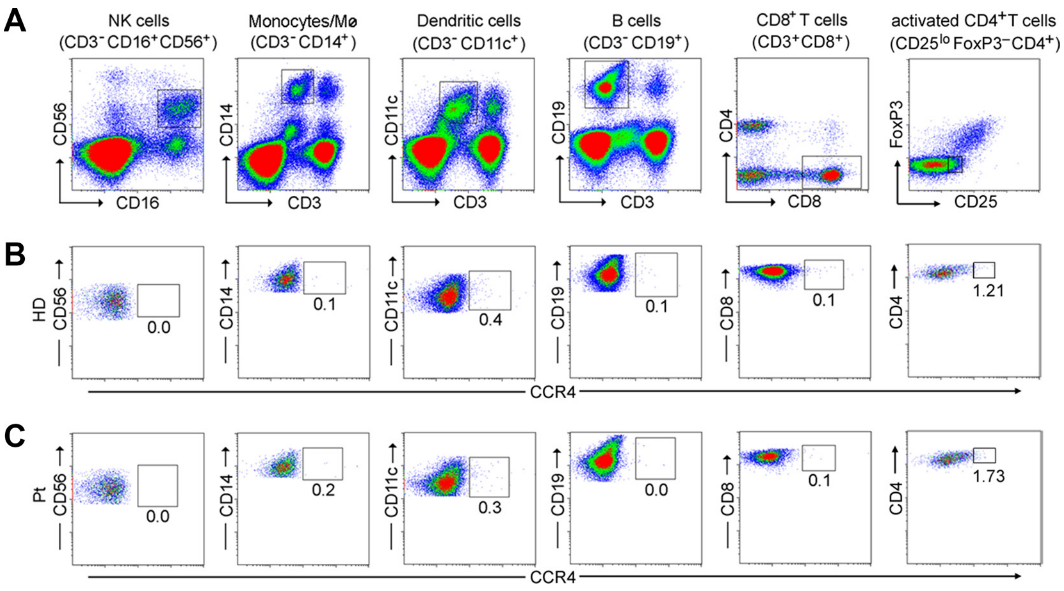

Supplementary Figure S4: CCR4 is not significantly expressed on NK cells, monocytes/macrophage, DCs, B cells, CD8⁺ T cells, and activated CD4⁺ T cells. (**A**) PBMCs from 13 HNSCC patients and 10 healthy donors were stained for cell type-specific markers. CCR4 expression in each cell population from 10 healthy donors (**B**) and 13 HNSCC patients (**C**) was analyzed by flow cytometry. Representative data from one HNSCC patient and one healthy donor are shown.

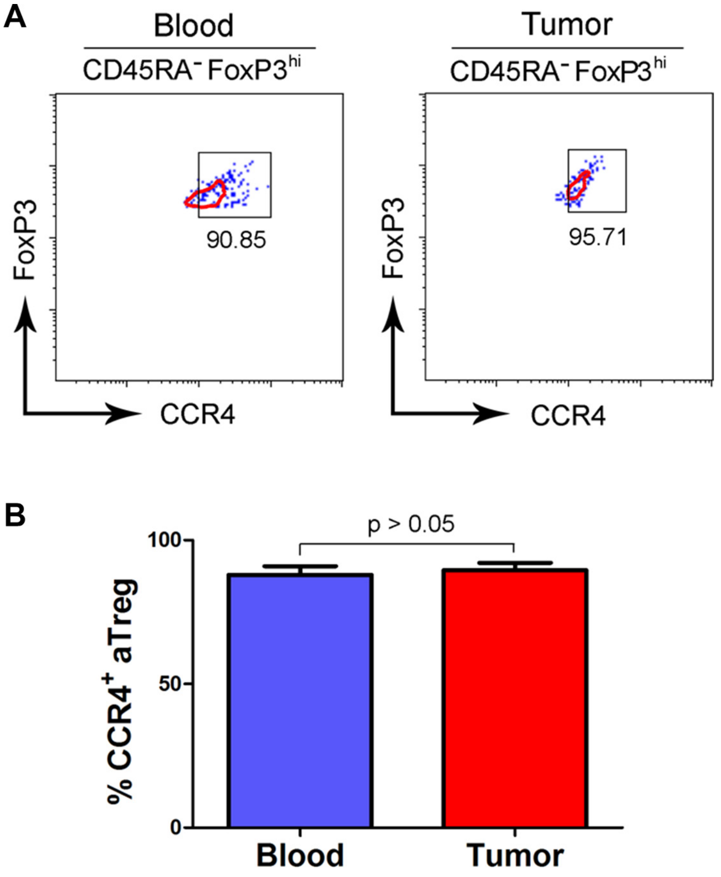

Supplementary Figure S5: CCR4 expression in tumor infiltrating aTreg cells corresponds to the expression in aTreg cells in blood. (**A**) CCR4 expression was analyzed in aTreg cells from tumor tissues and blood. Data for one representative patient are shown. (**B**) The statistical difference between two groups was analyzed using Mann-Whitney *U*-tests ($n = 18$).

Supplementary Table S1: Relationship between tumor-infiltrating aTreg cells and clinical variables

	Tumor grade				Stage		
	**Poor**	**Moderate**	**Well**	***P***	**I–II**	**III–IV**	***P***
aTreg cells (median, range)	4.50 (0.75–17.50)	4.00 (0–18.50)	2.88 (0–24.00)	> 0.05	2.50 (0–9.25)	9.75 (0–24.00)	< 0.001

Supplementary Table S2: Factors influencing survival in 72 LSCC patients as determined using the Cox proportional hazards model

	Overall survival	
	P	**Relative risk (95% CI)**
aTreg cells	0.035	4.05 (1.10–14.88)
Stage (I–II vs. III–IV)	0.040	1.65 (1.02–2.65)

LSCC: Laryngeal squamous cell carcinoma; CI: Confidence Interval.
